# Efficacy of StepAdd, a Personalized mHealth Intervention Based on Social Cognitive Theory to Increase Physical Activity Among Patients With Type 2 Diabetes Mellitus: Protocol for a Randomized Controlled Trial

**DOI:** 10.2196/53514

**Published:** 2024-02-23

**Authors:** Kayo Waki, Yuya Tsurutani, Hironori Waki, Syunpei Enomoto, Kosuke Kashiwabara, Akira Fujiwara, Kazuki Orime, Sho Kinguchi, Toshimasa Yamauchi, Nobuhito Hirawa, Kouichi Tamura, Yasuo Terauchi, Masaomi Nangaku, Kazuhiko Ohe

**Affiliations:** 1 Department of Biomedical Informatics Graduate School of Medicine The University of Tokyo Tokyo Japan; 2 Department of Diabetes and Metabolic Diseases Graduate School of Medicine The University of Tokyo Tokyo Japan; 3 Department of Planning, Information and Management The University of Tokyo Hospital Tokyo Japan; 4 Endocrinology and Diabetes Center Yokohama Rosai Hospital Yokohama Japan; 5 Department of Metabolism and Endocrinology Akita University Graduate School of Medicine Akita Japan; 6 Data Science Office Clinical Research Promotion Center The University of Tokyo Hospital Tokyo Japan; 7 Department of Nephrology and Hypertension Yokohama City University Medical Center Yokohama Japan; 8 Department of Endocrinology and Diabetes Yokohama City University Medical Center Yokohama Japan; 9 Department of Medical Science and Cardiorenal Medicine Yokohama City University Graduate School of Medicine Yokohama Japan; 10 Department of Endocrinology and Metabolism Yokohama City University Graduate School of Medicine Yokohama Japan; 11 Division of Nephrology and Endocrinology Graduate School of Medicine The University of Tokyo Tokyo Japan

**Keywords:** digital therapeutics, behavior change, social cognitive theory, exercise, type 2 diabetes mellitus, mobile app, randomized controlled trial, mobile phone

## Abstract

**Background:**

Increasing physical activity improves glycemic control in patients with type 2 diabetes (T2D). Mobile health (mHealth) interventions have been proven to increase exercise, but engagement often fades with time. As the use of health behavior theory in mHealth design can increase effectiveness, we developed StepAdd, an mHealth intervention based on the constructs of social cognitive theory (SCT). StepAdd improves exercise behavior self-efficacy and self-regulation through the use of goal-setting, barrier-identifying, and barrier-coping strategies, as well as automatic feedback functions. A single-arm pilot study of StepAdd among 33 patients with T2D showed a large increase in step count (mean change of 4714, SD 3638 daily steps or +86.7%), along with strong improvements in BMI (mean change of −0.3 kg/m^2^) and hemoglobin A_1c_ level (mean change of −0.79 percentage points).

**Objective:**

In this study, we aim to investigate the efficacy and safety of StepAdd, an mHealth exercise support system for patients with T2D, via a large, long, and controlled follow-up to the pilot study.

**Methods:**

This is a randomized, open-label, multicenter study targeting 160 patients with T2D from 5 institutions in Japan with a 24-week intervention. The intervention group will record daily step counts, body weight, and blood pressure using the SCT-based mobile app, StepAdd, and receive feedback about these measurements. In addition, they will set weekly step count goals, identify personal barriers to walking, and define strategies to overcome these barriers. The control group will record daily step counts, body weight, and blood pressure using a non–SCT-based placebo app. Both groups will receive monthly consultations with a physician who will advise patients regarding lifestyle modifications and use of the app. The 24-week intervention period will be followed by a 12-week observational period to investigate the sustainability of the intervention’s effects. The primary outcome is between-group difference in the change in hemoglobin A_1c_ values at 24 weeks. The secondary outcomes include other health measures, measurements of steps, measurements of other behavior changes, and assessments of app use. The trial began in January 2023 and is intended to be completed in December 2025.

**Results:**

As of September 5, 2023, we had recruited 44 patients. We expect the trial to be completed by October 8, 2025, with the follow-up observation period being completed by December 31, 2025.

**Conclusions:**

This trial will provide important evidence about the efficacy of an SCT-based mHealth intervention in improving physical activities and glycemic control in patients with T2D. If this study proves the intervention to be effective and safe, it could be a key step toward the integration of mHealth as part of the standard treatment received by patients with T2D in Japan.

**Trial Registration:**

Japan Registry of Clinical Trials (JRCT) jRCT2032220603; https://rctportal.niph.go.jp/en/detail?trial_id=jRCT2032220603

**International Registered Report Identifier (IRRID):**

DERR1-10.2196/53514

## Introduction

### Background

Worldwide, approximately 537 million people aged between 20 and 79 years have type 2 diabetes (T2D) [1]. T2D is a serious public health concern with a considerable impact on health expenditures and human life, causing 4.2 million deaths per year [2,3]. T2D is a disease that is difficult to cure once it develops, and if left untreated, it causes complications such as cardiovascular disease and microvascular complications [4].

Exercise has been proven to improve glycemic control and other metabolic parameters, including low-density lipoprotein cholesterol, visceral adipose tissue, and blood pressure (BP) [5]. Patients with T2D are recommended to commit to a minimum of 150 minutes of moderate to vigorous physical activity per week. As walking has been rated as the most favored form of physical activity by sedentary groups, it is a suitable form of exercise for patients with T2D, who typically have low physical activity [6,7]. The recommended 150 minutes of activity translates into taking at least 7500 steps per day, of which 3000 steps should be at moderate to vigorous intensity [8]. Although physical exercise has been proven to be effective in improving blood glucose levels, reducing cardiovascular risk factors, and improving cardiorespiratory fitness in patients with T2D, lack of motivation has been reported to be a barrier [9]. It is difficult for patients to change their current exercise behavior by themselves [9]. It has also been reported that adherence to physical activities is lower than adherence to medication regimens [10].

Mobile health (mHealth) interventions such as mobile phone apps that support self-management have proven to be effective in increasing physical activity of patients [11,12] and improving glycemic control [13,14]. According to the American College of Sports Medicine, web-based fitness programs, exercise-related digital services, and use of mobile phones are becoming increasingly popular in the worldwide exercise community [15]. Recent meta-analyses found a moderate to large positive effect in daily step changes owing to the use of smartphone apps that focus on physical activities [16,17]. Although mHealth is effective, patients lose motivation to engage with the intervention after some time [18,19]. Personalization of intervention components has been shown to increase patient motivation and engagement in using the app [20]. Several studies have found that tailored interventions are more effective in changing health behavior than nontailored interventions [21,22].

Although several studies evaluating the effectiveness of personalized interventions have reported an increase in the physical activity levels of users [23,24], so far, studies generally do not use a theory-based framework with mHealth. In particular, social cognitive theory (SCT), with a human agency model in which individuals proactively self-reflect, self-regulate, and self-organize, offers a powerful framework that could support improved mHealth interventions [25,26]. SCT has been used to understand behaviors relating to physical activity owing to its emphasis on the dynamic interactions between the individual, environment, and behavior [27]. SCT-based interventions were found to be effective in changing physical activity behaviors among people with prediabetes [28].

To address this research gap, we developed a personalized smartphone-based mHealth intervention, StepAdd, to improve physical activity levels and T2D control among patients with T2D by using the SCT framework. In 2021, we conducted a pilot study of StepAdd among 33 patients with T2D attending Mitsui Memorial Hospital in Japan, using a pre-post evaluation design over 12 weeks. The results show very high retention (97%), very large increase in mean daily steps (from 5436 to 10,150 steps per day; 86.7% increase), and positive changes in BMI (mean change of −0.3 kg/m^2^) and hemoglobin A_1c_ (HbA_1c_) levels (mean change of −0.79 percentage points) [29]. Growth in step count continued throughout the intervention, as did growth in step count goals. The step goal achievement rate remained steady and high throughout the intervention period. With strong positive impacts and strong ongoing engagement in this pilot study, we concluded that StepAdd warranted a deep study.

This trial aims to investigate the efficacy of StepAdd, also known as diabetic kidney disease–exercise therapy, in a long (24 weeks), randomized controlled trial. The specific research objectives and hypotheses are as follows.

### Objective 1

The first objective is to investigate the efficacy of StepAdd in improving physical activity levels, as measured using daily step count.

We hypothesize that, at the end of the intervention, the intervention group will achieve a statistically significant increase in daily step count relative to the counts of the control group.

### Objective 2

The second objective is to investigate the efficacy of StepAdd in reducing HbA_1c_ levels.

We hypothesize that, at the end of the intervention, the intervention group will achieve a statistically significant reduction in the primary outcome, HbA_1c_ levels, relative to the HbA_1c_ levels in the control group.

### Objective 3

The third objective is to assess the effect of StepAdd in improving 7 self-care behaviors (walking duration, adherence to T2D self-care, self-management behavior to enhance and maintain physical activity, self-regulation of physical activity, self-efficacy to achieve targeted daily steps, self-efficacy to deal with barriers to achieving the targeted daily steps, and self-efficacy for health-promoting behaviors) and 10 health indicators (fasting blood glucose, estimated glomerular filtration rate, BMI, BP, high-density lipoprotein, low-density lipoprotein, triglyceride, locomotive syndrome, T2D-related emotional distress, and T2D-dependent quality of life).

We hypothesize that, at the end of the intervention, the intervention group will achieve a statistically significant improvement in the 7 self-care behaviors and 10 health indicators.

## Methods

### Study Design

**Figure 1:**
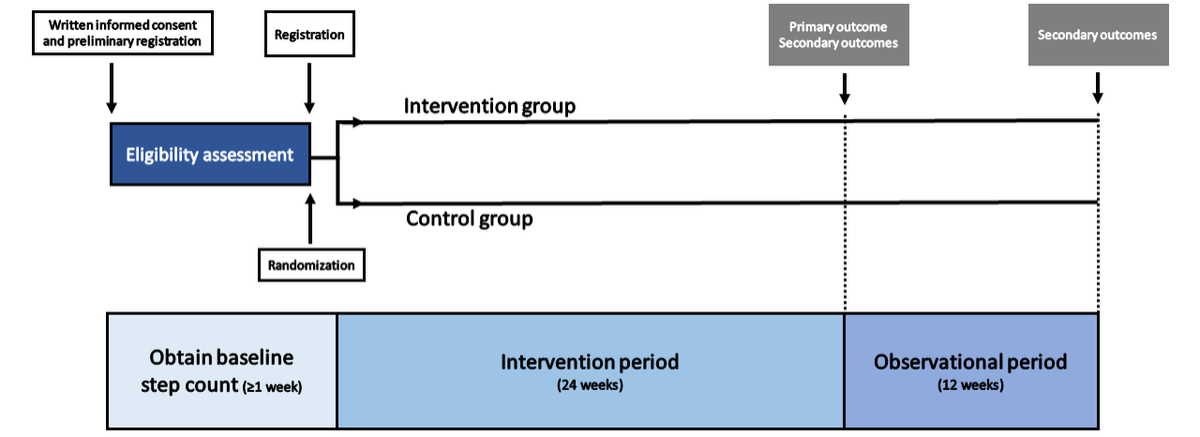
Overview of study timeline—at least 1 week of baselining, 24 weeks of intervention, and 12 weeks of observation.

This study is an open-label, multicenter (Textbox 1), confirmatory, 2-armed, randomized controlled trial to study the efficacy of an mHealth intervention, StepAdd, in promoting exercise behavior among patients with T2D. The conduct and reporting of the trial will follow the CONSORT (Consolidated Standards of Reporting Trials) guidelines. This study (Figure 1) begins with recruitment, a baseline measurement period (nominally 2 weeks, but at least 7 days), and randomized assignment to the intervention and control groups. The patients in the intervention group will use the SCT-based StepAdd smartphone app to measure body weight (BW), BP, and daily steps; receive personalized feedback; and develop updated step goals. The patients in the control group will use a placebo smartphone app that simply measures and reports BW, BP, and daily steps. Both groups will receive monthly consultations with their physician. The intervention will last for 24 weeks and will be followed by a 12-week observational period. This study will be conducted over 3 years, from January 1, 2023, to December 31, 2025.

**Participating institutions—5 medical centers within Japan.**

- University of Tokyo Hospital
- Akita University Hospital
- Yokohama City University Hospital
- Yokohama City University Medical Center
- Yokohama Rosai Hospital

### Intervention Design

**Figure 2:**
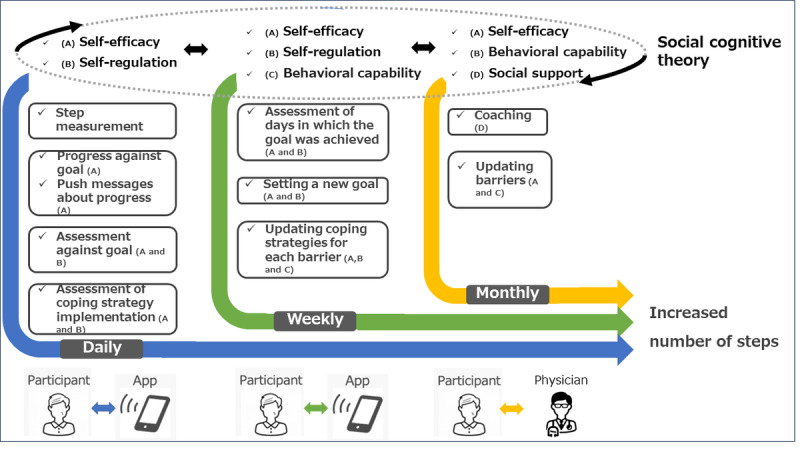
StepAdd’s process for implementing social cognitive theory (SCT)–based personalization on daily, weekly, and monthly timelines (adapted from Wei Thing Sze et al [29], Copyright 2023, used with permission from Elsevier).

This mHealth intervention uses a pedometer; a body weighing scale; a BP monitor; and a smartphone with an mHealth app, StepAdd. The app is an iOS app on an iPhone that communicates with the StepAdd server. An administrative screen is used by a medical professional (either a physician or a clinical research coordinator who is overseen by a physician). Pedometer count, BP, and BW are measured at home. The pedometer count is synchronized with the app continuously during the day, and the BP and BW are synchronized with the app daily when patients open the app at the time of measurement. The app interacts with the patient to establish personalized goals, provide personalized feedback, and define and assess the efficacy of patient-specific coping skills (Figure 2 [29]). This app was developed in collaboration with foo.log Co Ltd, a software development company. The control group uses a placebo app supporting the same measurements but without personalized goals and feedback (Table 1).

**Table 1:** Features of the trial’s StepAdd and placebo apps and additional features provided to the intervention group.

Features	StepAdd app	Placebo app
Display, record, and upload step count, BW^a^, and BP^b^	Yes	Yes
Reminders to measure BW and BP and to carry pedometer	Yes	Limited reminders to carry pedometer
Review of step count, BW, and BP	Yes	No
Exercise instructions	Yes	No
Goal setting	Yes	No
Coping strategies to overcome barriers to achieving step goals	Yes	No
Reminders about coping strategies	Yes	No
Display of recorded health data that were registered by physicians from the administrative screen	Yes	No
Daily reflection assistance	Yes	No
Weekly reflection assistance	Yes	No
Record about whether the tasks agreed with the health care professional are completed	Yes	No
Personalized feedback	Yes	No

^a^BW: body weight.

^b^BP: blood pressure.

The intervention uses the framework of SCT [25,26]. The intervention incorporates the SCT constructs that are most used in physical activity research: self-efficacy, self-regulation, behavioral capability, and social support [30]. Self-efficacy is an individual’s belief in their ability to perform a particular behavior successfully. Individuals are less likely to engage in a behavior if they do not believe they are capable of producing the desired effects. Sources of self-efficacy include mastery experience (the successful accomplishment and mastering of a desired behavior) and verbal persuasion (receiving verbal encouragement from others for performing a behavior). Self-regulation, one of the constituent concepts of SCT that is closely linked with behavior change, is a dynamic feedback loop in which an individual sets a target goal and, by comparing this goal with their present state, is motivated to change their behavior to achieve the goal [31]. Motivation does not originate from the goals themselves but from the individual analyzing their own behavior with reference to their goal [32]. Self-regulation includes behaviors such as setting personal goals and planning courses of action to achieve them, and it is often motivated by expected positive outcomes. Behavioral capability is a person’s actual ability to perform a behavior through essential knowledge and skills. Social support involves identifying others who will provide encouragement in the form of moral support, participation in the behavior, and accountability [33].

SCT constructs integrated in this app focused on a self-regulated walking habit using goal setting and perceived self-efficacy [34]. Users set small, progressive, and realistic goals for daily step counts (self-efficacy and goal setting), track and monitor daily step count performance (self-observation), and evaluate their own progress against the goals (self-judgment). The belief that one’s progress is acceptable, along with the anticipated satisfaction of meeting a goal, enhances self-efficacy and motivation (self-reaction) [34]. The intervention provides social support through monthly meetings with a physician who examines the use of the app and provides advice, verbal encouragement, and support regarding the participants’ exercise behavior (behavioral capability and social support).

The app proposes the target number of daily steps for the following week according to the achievement of the current week’s step count against goals. Our algorithm [29] increases, maintains, or decreases the suggested goal based on the number of days in the previous week in which the user achieved their goal. Increases are either 300 or 500 steps, depending on past goal achievement levels. The patient may choose to decrease their goal by decrements of 200 steps if they are not confident in being able to complete the goal for ≥5 days. In this way, the app recommends goal adjustments according to the user’s self-efficacy in goal achievement. Personalized goal setting also enhances self-regulation through forethought (setting a goal and deciding on goal strategies), performance control (using goal-directed actions and monitoring performance), and self-reflection (evaluating one’s goal progress and adjusting the strategies to ensure success) [35].

**Figure 3:**
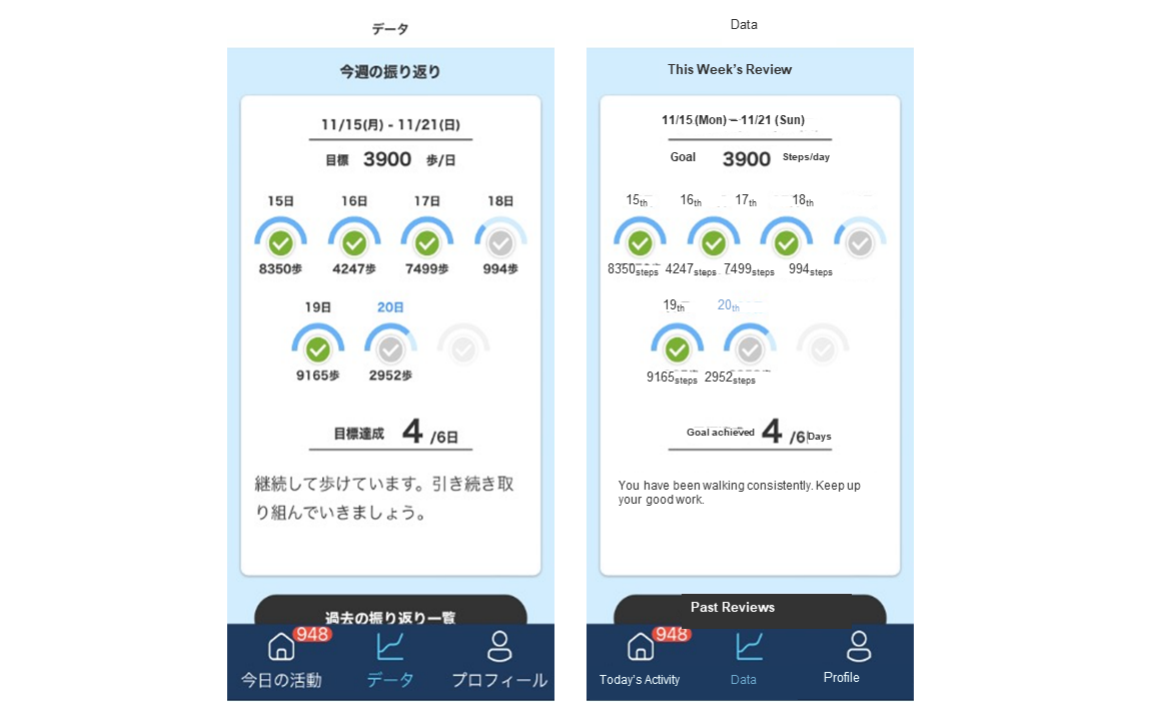
Example of end-of-week feedback (with English translation) showing daily and weekly performance against goals.

The app provides automated feedback with individualized advice. Status against the daily goal is available continuously on the app. Self-regulation is enhanced as patients are able to self-monitor their behavior through the personalized feedback. The feedback also enhances self-efficacy as it acts as a means of verbal persuasion, which is a source of self-efficacy [25,26]. The app automatically communicates with the patients through push notifications 4 times a day (at 11 AM, 1 PM, 4 PM, and 6 PM) to communicate the achievement against the step goals. For example, if the target number of steps have not been reached at 11 AM, the message “The number of steps registered by 11 o’clock is [participants’ registered number of steps]. The additional number of steps required is [target number of steps minus current number of steps] to achieve your goal” appears on the app. The day’s achievement against the goal is shown at 6 PM. If the participant has reached the target step goal, the message “Today’s steps have significantly exceeded your goal! Congratulations. You’re in good shape tomorrow. Don’t get tired” appears on the app. At the end of the week, the app provides feedback by displaying the number of days in the week that the step goals were achieved (Figure 3).

**Figure 4:**
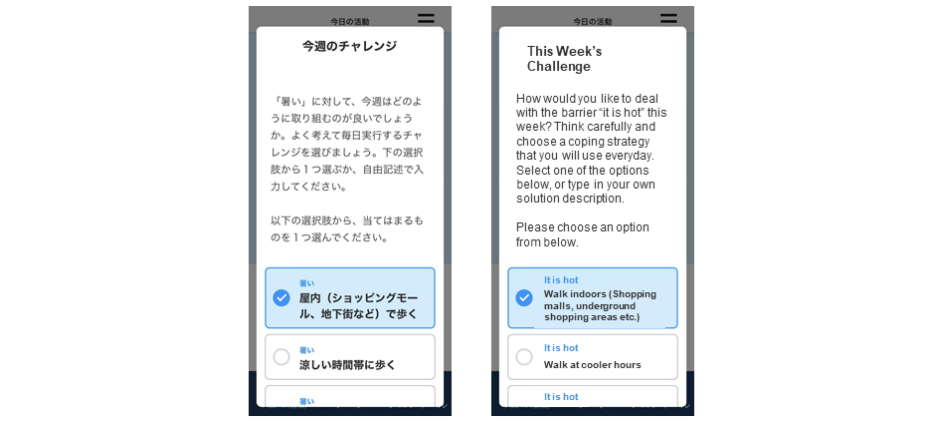
Example of personalized coping planning strategy on the app (with English translation) showing how patients select the strategy to use for the upcoming week.

Individualized coping planning enhances self-regulation by allowing patients to evaluate the possible barriers to achieving their step goals. Each week, participants select or input 1 to 3 barriers that prevented them from achieving the targeted number of steps, and for each barrier, they suggest a coping strategy that could be useful to overcome the barrier (Figure 4). The coping strategies can be selected from a literature-based list of solutions to common barriers to walking [36-39] or can be freely described by the participants. At the end of each day, participants use the app to report about how many coping strategies they implemented that day. At the end of each week, the app provides feedback about the coping strategy use by displaying the average number implemented per day.

### Patient Recruitment

Patients will be recruited at 5 medical institutions in Japan: University of Tokyo Hospital, Akita University Hospital, Yokohama City University Hospital, Yokohama City University Medical Center, and Yokohama Rosai Hospital. Recruitment will be conducted by attending physicians during patients’ regular consultations at outpatient clinics. Recruitment will be conducted over 2 years, starting from January 1, 2023, and ending on December 31, 2024.

To detect 0.35% difference in the primary outcome (change in HbA_1c_ level), assuming an SD of 0.74% as seen in a previous study [40], and to achieve a 2-sided significance level of .05 and a statistical power of 80%, the minimum sample size is 72 patients per group [41]. On the basis of an assumed dropout rate of 10%, we are targeting to recruit a total sample of 160 patients (n=80, 50% in the intervention group; n=80, 50% in the control group).

All participants will receive a thorough written and verbal explanation about participation. We will subsequently obtain written informed consent from all study participants before screening for eligibility to participate in the study. If findings regarding efficacy and safety that may affect patient consent are discovered at any points during the trial, we will swiftly disclose this information to participants and acquire their renewed consent.

To focus on patients who are likely to benefit and likely to be capable of participating, we will recruit patients who meet all the inclusion criteria (Textbox 2). To ensure that patients can safely and effectively participate in the intervention, we will recruit patients who do not meet any of the exclusion criteria (Textbox 3). We will use a transtheoretical model (TTM) questionnaire to categorize the participants who are currently at the contemplation stage (willing to change health behavior within the next 6 months), preparation stage (willing to change health behavior within the next month), or action stage (has made modifications to health behavior) [42]. In our previous study, patients who were in the contemplation and preparation stages of TTM were more likely to prefer to use smartphone-based self-management tools than those in the precontemplative stage [43]. Therefore, we targeted patients who are in the contemplation, preparation, or action stages of achieving the target goal of 10,000 steps a day.

We will provisionally register patients who meet the eligibility criteria. We will then provide them with a step-counting device and a smartphone with just the step-counting part of the placebo app. We will ask patients to record their daily step count for a baseline period (nominally 2 weeks, but at least 7 days), and then, we will measure their HbA_1c_ level at the end of the screening period. We will enroll patients who were able to measure their step count for ≥7 days during the baseline period (to ensure that we study patients who are able to adequately participate in the study) and who continue to have an HbA_1c_ level ≥7.5% (to ensure that patients continue to have high HbA_1c_ level after the screening period). We believe that the 2-week trial will have no significant effect on outcomes. We will then use the electronic data records to randomize eligible participants in 1:1 ratio to either the intervention group or the control group using the covariate-adaptive randomization by minimization method to ensure covariance balance for age (<65 years and ≥65 years), sex, HbA_1c_ level (<8.5% and ≥8.5%), and institution [44].

**Inclusion criteria of the study, focusing on patients who are likely to benefit from the intervention and are likely to be capable of participating.**

**Inclusion criteria**

- Diagnosed with type 2 diabetes mellitus
- Being in the contemplation, preparation, or action stages of the transtheoretical model to achieve the target step count of 10,000 steps per day
- Hemoglobin A_1c_ level ≥7.5% and ≤10% at the time of consent acquisition
- No change in antidiabetic medication within 8 weeks before the time of consent acquisition
- Aged ≥18 years
- Systolic blood pressure <180 mm Hg and diastolic blood pressure <110 mm Hg at the time of consent acquisition
- eGFR ≥45 mL/min/1.73 m_2_ recorded at least once in the 12 weeks before the time of initial registration (V0)
- Urine albumin-creatinine ratio is <300 mg/gCr on at least 1 occasion in the past year (52 weeks) before V0
- BMI ≥22 kg/m_2_
- No incidences of severe hypoglycemic attacks where assistance by others was required in the 12 weeks before V0
- No symptoms of a possible hypoglycemic attack (including palpitations, tremors, dizziness, lightheadedness, anxiousness, loss of consciousness, sweating, pale face, tachycardia, headache, drowsiness, blurred vision, and convulsion) observed in the 12 weeks before V0
- Able to attend consultations at designated times during the trial
- Fully informed about participation in this study and has given free and voluntary written consent based on thorough understanding of the study

**Exclusion criteria, focusing on patients who may not be able to participate safely or whose participation may interfere with the effectiveness of the study.**

**Exclusion criteria**

- Walked an average of ≥10,000 steps a day in the 4 weeks before V0 (must be verifiable by pedometer, etc)
- Wearing a pacemaker
- Using continuous glucose monitoring (but do not exclude if self-monitoring of blood glucose level or intermittently scanned continuous glucose monitoring was conducted ≥8 weeks before V0)
- Diagnosed with hyperthyroidism and have received treatment other than thyroid hormone replacement in the year before V0
- Diagnosed with a moderate to severe heart condition that requires exercise restriction as assessed by a physician
- Require exercise treatment restrictions as assessed by a physician at V0—decision made with reference to “Cases in which exercise therapy should be prohibited or restricted” in the Guidelines for Diabetes Treatment (Japan Diabetes Society; 2022-2023)
- Hemoglobin <10 g/dL in the 12 weeks before V0
- Serum albumin ≤3 g/dL in the 12 weeks before V0
- Diagnosed with preproliferative retinopathy or retinopathy of a later stage within the year before V0
- Cannot undergo exercise treatment
- Pregnancy, including any possibility or intention of pregnancy
- Participation in other trials at the time of initial registration (V0)
- Impaired cognitive function, as determined by the investigator or subinvestigator
- Any other reason that the patient is classified as unfit for participation by the investigator or subinvestigator (a record must be maintained about the reason the patient was determined to be ineligible)

### Intervention Process

The study (Table 2) includes 10 events (V0-V9), a 24-week continuous measurement phase (M), and a 12-week follow-up observation phase. Initial measurements will be made at event V0. At V0, we will provide each participant with a near field communication–enabled pedometer (A&D; UW-204NFC) and an iPhone (iPhone 8 with iOS 16) prepared with the placebo app configured for step count only. We will direct the participants to wear the pedometer throughout the day except during sleep. We will use the step count gathered during V1 to confirm eligibility and to set a step count baseline.

The surveys that will be used in this study are listed in Textbox 4.

**Table 2:** Study events—1 measurement period and 10 visits spanning approximately 37 weeks.

Event	Focus	Time	Key activities
V0	Physical baseline assessments and initial registration	Enrollment—≥7 days before the beginning of the intervention	Collect consent Collect demographic information: date of birth, sex, smoking and drinking status, medical history, diet and exercise habit, household living condition, employment, use of smartphones, habit of step count measurement, and stage of behavior change according to the transtheoretical model of change regarding walking 10,000 steps a day Collect physical data: blood tests to measure HbA_1c_^a^, FBG^b^, LDL^c^ cholesterol, HDL^d^ cholesterol, TG^e^, and eGFR^f^; urinalysis; and height, body weight, and BP^g^ measurements Collect medication and combination therapy status Assess health literacy Conduct surveys (Textbox 4) Distribute pedometer and placebo-equipped phone
V1	Step count baseline	From V0 to the day before V2 (at least 7 days)	Patients record daily steps and determine step count baseline
V2	Registration, allocation, and beginning of the intervention	Beginning of the intervention (end of week 0)	Measure HbA_1c_ level Finalize registration and allocation Distribute BP monitor and scale Activate all placebo and StepAdd features Collect medication and combination therapy status Check for adverse events
M	Ongoing measurement	From V2 to V8	Daily measurements via app Intervention only: daily feedback, weekly feedback, and weekly personalized goal setting via app Check for defects
V3	Hospital visit 1	End of week 4	Check app use and equipment Check for adverse events Intervention: coach goals and coping strategies Collect medication and combination therapy status
V4	Hospital visit 2	End of week 8	Check app use and equipment Check for adverse events Intervention: coach goals and coping strategies Collect medication and combination therapy status
V5	Interim hospital visit	End of week 12	Collect physical data: blood tests to measure HbA_1c_, FBG, LDL cholesterol, HDL cholesterol, TG, and eGFR; urinalysis; and body weight and BP measurements Check app use and equipment Check for adverse events Intervention: coach goals and coping strategies Collect medication and combination therapy status Conduct surveys (Textbox 4)
V6	Hospital visit 4	End of week 16	Check app use and equipment Check for adverse events Intervention: coach goals and coping strategies Collect medication and combination therapy status
V7	Hospital visit 5	End of week 20	Check app use and equipment Check for adverse events Intervention: coach goals and coping strategies Collect medication and combination therapy status
V8	End of the intervention	End of week 24	Collect physical data: blood tests to measure HbA_1c_, FBG, LDL cholesterol, HDL cholesterol, TG, and eGFR; urinalysis; and body weight and BP measurements Collect medication and combination therapy status Check for adverse events Conduct surveys (Textbox 4) Assess usefulness and usability
V9	End of follow-up	End of week 36	Collect physical data: blood tests to measure HbA_1c_, FBG, LDL cholesterol, HDL cholesterol, TG, and eGFR; urinalysis; and body weight and BP measurements Collect medication and combination therapy status Check for adverse events Conduct surveys (Textbox 4)

^a^HbA_1c_: hemoglobin A_1c_.

^b^FBG: fasting blood glucose.

^c^LDL: low-density lipoprotein.

^d^HDL: high-density lipoprotein.

^e^TG: triglyceride.

^f^eGFR: estimated glomerular filtration rate.

^g^BP: blood pressure.

**Surveys—9 surveys assessing type 2 diabetes–related behaviors and outcomes.**

**Problem Areas in Diabetes**

- 20 items to assess type 2 diabetes–related emotional distress [45]

**Locomo 25**

- 25-question risk assessment to evaluate musculoskeletal disorders such as walking disability, difficulty in daily living, or pain in the body [46]

**Evaluation Scale for Self-Management Behavior Related to Physical Activity of Type 2 Diabetic Patients**

- Assessments of the following:
  Self-management behavior to enhance daily physical activity
  Self-management behavior to maintain the level of physical activity [47]

**Steps achieved**

- Self-Efficacy Scale relative to achieving the targeted daily steps [48]

**Dealing with barriers to achieving the targeted daily steps**

- Self-Efficacy Scale relative to dealing with barriers to achieving the targeted daily steps [49]

**Physical Activity Self-Regulation Scale–12; Japanese version**

- Addresses self-monitoring, goal setting, eliciting social support, reinforcement, time management, and relapse prevention
- Rated from 1 (never use strategy) to 5 (use strategy very often)
- Scores are the sum of the 2 individual item scores and summed self-regulation subscale scores for a measure of overall self-regulation [50]

**The Summary of Diabetes Self-Care Activities Measure; Japanese version**

- Assesses type 2 diabetes self-care activities [51]

**Health behavior “active coping behavior with disease”**

- Self-Efficacy Scale for health behavior “active coping behavior with disease” subscale [52]

**Audit of Diabetes-Dependent Quality of Life; Japanese version**

- Assesses type 2 diabetes–dependent quality of life [53]

Following randomization, we will begin the intervention. We will provide participants a Bluetooth-enabled sphygmomanometer (A&D; UA-651BLE Plus) and a Bluetooth-enabled weighing scale (A&D; UC-352BLE; in addition to the already provided pedometer and phone). For the control group, we will enable BP and BW measurement functions in the placebo app. For the intervention group, we will replace the placebo app with StepAdd and define the initial goals and barriers. Both the intervention and control groups will watch a video regarding lifestyle modification, self-management of T2D, and effect of exercise on glycemic control.

The main part of the intervention is the daily measurement by all patients throughout phase M. We will direct participants to measure their BW and BP daily upon awakening and wear the pedometer throughout the day except during sleep. All the participants will log the measurement data by syncing the recording devices (weighing scale, sphygmomanometer, and pedometer) to the app, rather than by manually inputting the data. These data are then uploaded to the server and made visible to health care professionals via the administrative screen. Intervention group participants receive personalized feedback about their step count performance 4 times a day. At the end of each week, intervention group participants review their weekly step count performance on the app, determine their personal barriers to achieving the step goals, and identify the possible coping strategies. StepAdd then provides a personalized step goal recommendation for the following week.

Every 4 weeks (events V3-V7), both groups will meet with a physician who will advise patients regarding lifestyle modifications and use of the app and, for the intervention group only, help with goals and coping strategies. Baseline measurements (other than collection of some background information) are repeated at the interim (V5), at the end of the intervention (V8), and at the end of the follow-up observation period (V9). We will collect the equipment at V8, before the follow-up observation period. The purpose of the observational period is to assess whether the intervention-induced effects are maintained after the end of the intervention.

During the study, we will not restrict the use of supplements and drinks that can affect blood glucose level and BP. In both the intervention and control groups, T2D treatment details, such as changes or additions of oral T2D medications, insulin, and glucagon-like peptide–1 receptor agonists and changes in medication dosage, can be changed at the discretion of the attending physician.

If patients discontinue the trial or stop using the app, we will define this as discontinuation, and we will collect the measurements within 7 days of this discontinuation. If participation in the study is discontinued during the intervention period, we will collect the V8 measurement items. If participation in the study is discontinued during the observation period, we will collect the V9 measurements items, except for urinalysis.

### Monitoring, Quality Control, and Data Management

An auditor who is independent from the departments involved in the trial, including those responsible for monitoring, will inspect the medical institution and other facilities involved to ensure that the trial is conducted appropriately.

### Ethical Considerations

This trial will be conducted in compliance with the Declaration of Helsinki, Pharmaceutical and Medical Device Act, Ministerial Ordinance on Good Clinical Practice for Medical Devices, and all other guidelines in relation to these regulations (jRCT2032220603). This study was approved by the institutional review board of the University of Tokyo School of Medicine (approval number 2022012-11DY). We will obtain written informed consent from all study participants. All patient data will be anonymized. Patients will be compensated JPY 7000 (approximately US $50) for each visit to the hospital.

### Outcome Measures

#### Primary Outcome

The primary outcome of this study is the between-group differences in the change of HbA_1c_ value between baseline (V2) and either the end of the intervention period (week 24 [V8]) or, for patients who discontinue before V8, the point of discontinuation. Reduction of HbA_1c_ level by 0.35 percentage points is considered to be clinically significant [54,55].

#### Secondary Outcomes

Secondary outcomes (Table 3) include health measures, measurements of steps, measurements of other behavior changes, and assessments of app use. We will also assess safety outcomes such as hypertension requiring medical assistance (assessed from the system records), subjective hypoglycemia, pain in the lower back and lower extremities (tarsus, thighs, knees, calves, shins, ankles, and feet), and any other adverse events (all assessed through patient interviews).

**Table 3:** Secondary outcomes investigated in the study—22 areas spanning health outcomes, exercise outcomes, other behavior changes, and app assessments.

Categories and item numbers		Outcomes
**Health outcomes**		
	1	HbA_1c_^a^ level (%) Change in HbA_1c_ level^b^ Proportion of patients with HbA_1c_ level <7%^c^
	2^d^	FBG^e^ (mg/dL) >5 hours after a meal
	3^d^	eGFR^f^ (L/min/1.73m2)
	4^d^	BMI (kg/m2)
	5^g^	Body weight measured at home (kg)
	6	Systolic and diastolic BP^h^ (mm Hg) BP measured at hospital^d^ BP measured at home^g^
	7^d^	HDL^i^ (mg/dL), LDL^j^ (mg/dL), and TG^k^ (mg/dL)
	8^l^	Concomitant medication intake (type of medication and daily dosage or weekly dosage for weekly formulation) Change in type 2 diabetes medication intake (either increased, unchanged, or reduced) Introduction of new medications to treat type 2 diabetes, hypertension, or dyslipidemia
	9^d^	Type 2 diabetes–related emotional distress (assessed using Problem Areas in Diabetes) [45]
	10^d^	Type 2 diabetes–dependent quality of life (assessed using Audit of Diabetes-Dependent Quality of Life) [53]
**Exercise outcomes**		
	11^g^	Daily step count
	12^d^	Walking duration
**Other behavior changes**		
	13^d^	Locomotive syndrome (assessed using Locomo 25) [46]
	14^d^	Type 2 diabetes self-care, assessed using the Japanese version of the Summary of Diabetes Self-Care Activities Measure [51]
	15^d^	Self-management behaviors related to physical activity, assessed using Evaluation Scale for Self-Management Behavior Related to Physical Activity of Type 2 Diabetic Patients [47]
	16^d^	Self-regulation of physical activity, assessed using the Japanese version of Physical Activity Self-Regulation Scale–12 [50]
	17^d^	Changes in walking self-efficacy: Self-efficacy for achieving the targeted daily steps [48] Self-efficacy for dealing with barriers to achieving the targeted daily steps (assessed using Self-Efficacy Scale of Walking Behavior) [49]
	18^d^	Changes in self-efficacy in health-promoting behavior, assessed using Positive Coping Behavior Towards Illness subscale within Self-Efficacy Scale for Health Promoting Behaviors [52]
	19^m^	Health literacy, assessed using Health Literacy Scale 14 [56]
**App assessments**		
	20^n^	App use is assessed through the following: Recording rate of body weight, step count, and BP and its change^o^ Goal achievement rate^p^ Goal increment rate^q^ Goal reduction rate^r^ Average number of coping planning strategies implemented per day^s^ Number of barrier identifications^t^
	21^u^	System feasibility and usability (only for the intervention group)
	22^v^	Number of emails, SMSs, and calls to encourage registration

^a^HbA_1c_: hemoglobin A_1c_.

^b^Includes both differences between week 0 (registration at V2) and week 12 (V5; or point of discontinuation, if earlier) and between week 0 (registration at V2) and week 36 (V9; or point of discontinuation, if earlier), which are compared within group and between groups, and includes the difference between week 0 (registration at V2) and week 24 (V8; or point of discontinuation, if earlier), which are compared within group.

^c^Includes all 3 proportions that are compared between groups: latest HbA_1c_ level up to week 12 (V5; or point of discontinuation, if earlier) <7%, latest HbA_1c_ level up to week 24 (V8; or point of discontinuation, if earlier) <7%, and latest HbA_1c_ level up to week 36 (V9; or point of discontinuation, if earlier) <7%.

^d^Includes all 3 differences: between provisional registration (V0) and week 24 (V8; or point of discontinuation, if earlier), between provisional registration (V0) and week 12 (V5; or point of discontinuation, if earlier), and between provisional registration (V0) and week 36 (V9; or point of discontinuation, if earlier), which are compared between groups.

^e^FBG: fasting blood glucose.

^f^eGFR: estimated glomerular filtration rate.

^g^Includes differences between the beginning of the intervention (V2; or from baseline period [V1] for steps) and week 4 (V3) and differences between each visit and each subsequent visit, which are compared between groups. In addition, they include measurements at each period (between visits), which are to be compared between groups. Item 11 also includes difference between baseline (V1) and 2 weeks before week 12 (V5; or point of discontinuation, if earlier) and week 24 (V8; or point of discontinuation, if earlier), which are compared within group and between groups.

^h^BP: blood pressure.

^i^HDL: high-density lipoprotein.

^j^LDL: low-density lipoprotein.

^k^TG: triglyceride.

^l^Includes assessment at week 12 (V5), week 24 (V8), and week 36 (V9; or point of discontinuation, if earlier), respectively, and it will be compared between groups.

^m^Will be assessed at provisional registration (V0).

^n^Includes comparisons of the differences over the first period of the intervention (from week 0 [V2] to week 4 [V3]) and differences over each subsequent period (between subsequent visits), which are compared between groups (only the recording rate of body weight, step count, and BP and its change) and within group (all assessments).

^o^Formula: (number of days in which measurements for BW, step count, and BP are recorded/total number of days) × 100.

^p^Formula: (number of days in which step goal was achieved/total number of days) × 100; examined only for the intervention group.

^q^Formula: (number of days in which step goal was increased/total number of reset times) × 100; examined only for the intervention group.

^r^Formula: (number of days in which step goal was reduced/total number of reset times) × 100; examined only for the intervention group.

^s^Formula: total number of coping strategies implemented over the period/total number of days; examined only for the intervention group.

^t^Number of barriers identified every 4 weeks; examined only for the intervention group.

^u^Will be assessed for the intervention group only, at week 24 (V8; or point of discontinuation, if earlier).

^v^Will be assessed at week 24 (V8; or point of discontinuation, if earlier).

### Statistical Analysis

In this study, we define 3 analysis populations: full analysis set (FAS), per protocol set (PPS), and safety analysis set (SAS). FAS is the set of all patients whose data were obtained at least once after randomization. We will follow the intention-to-treat principle and analyze data based on the assigned group in the FAS analysis. PPS is the FAS population with the exclusion of patients who were found to have violated the eligibility criteria after randomization or who did not use the app over a 3-week period. SAS is the set of all patients who used the StepAdd app or the placebo app at least once after randomization. In the safety analysis using SAS, we will analyze data based on the apps actually used by the patients, regardless of allocation. AS will be the primary analysis population, and PPS will provide supportive results. All safety analyses will be conducted in SAS.

Data about patients’ characteristics will be presented as mean, SD, minimum, 25th percentile, median, 75th percentile, and maximum for continuous variables and as frequency and proportion for categorical variables.

In the primary analysis, we will perform between-group comparison of the change in HbA_1c_ level from week 0 (V2) to week 24 (V8; or point of discontinuation, if earlier) using analysis of covariance, including baseline HbA_1c_ level (ie, HbA_1c_ level at week 0 [V2]) as a covariate in FAS. If participants discontinue the study, the latest measurement of HbA_1c_ level before V8 will be used.

We will also conduct 2 subgroup analyses with one classification by HbA_1c_ level at week 0 (V2; either <8.5% or ≥8.5%) and the other classification by BMI at the time of initial registration (V0; either <25 kg/m^2^ or ≥25 kg/m^2^). We will conduct subgroup analysis on the primary outcome in each analysis set to investigate whether the efficacy of StepAdd is consistent between these subgroups.

In the secondary analysis, the change in HbA_1c_ level at week 12 (V5) and week 36 (V9) will be analyzed as in the primary analysis. The proportion of patients with HbA_1c_ level <7% at week 12 (V5), week 24 (V8), and week 36 (V9; or point of discontinuation, if earlier) will be compared between the groups using Fisher exact test. The change from baseline in the step counts, various laboratory test values, and questionnaire scores at week 12 (V5), week 24 (V8), and week 36 (V9; or point of discontinuation, if earlier) will be analyzed as the primary end point. Changes in T2D medications, classified as weakened, unchanged, or strengthened, will be compared between groups using the Cochran-Mantel-Haenszel test. The proportion of new medications added will be compared between groups using Fisher exact test. Change in app data (step counts, BW, and BP) and their recording rates will be evaluated between each visit based on the week-0 (V2) to week-4 (V3) period and compared between groups using 2-tailed *t* tests. The McNemar test will be used for within-group comparison of the proportion of people who walked ≥10,000 steps in the 2 weeks before the intervention and the 2 weeks before week 24 (V8). A paired *t* test will be used for within-group comparison over the week-0 (V2) to week-4 (V3) period for goal achievement rate, goal increment rate, goal reduction rate, average number of coping planning strategies implemented per day, and number of barrier identifications.

As an exploratory analysis, we will use linear regression to investigate the relationship between the average number of coping strategies implemented per day and improvement in HbA_1c_ level in the intervention group. The analysis will use the average number of coping strategies implemented per day and baseline HbA_1c_ level as explanatory variables and the change in HbA_1c_ level as the response variable. The change from baseline to week 12 (V5), week 24 (V8), and week 36 (V9; or point of discontinuation, if earlier) will be evaluated.

We will conduct statistical analysis of safety outcomes. We will compare the proportion of incidences of hypoglycemia, joint pain, significantly elevated BP (≥145/95 mm Hg), and exacerbations of joint pain between groups using Fisher exact test. Other adverse events will be recorded using the Japanese version of the Medical Dictionary for Regulatory Activities categories for Preferred Term and System Organ Class, and their incidence proportions will be compared between groups using Fisher exact test. The severity of each incidence will be categorized into 3 grades (low, moderate, and high), and their between-group comparison will be conducted using the Cochran-Mantel-Haenszel test.

## Results

Recruitment began on January 1, 2023. As of September 5, 2023, we have recruited 44 patients. We expect the trial to be completed by October 8, 2025, with the follow-up observation period being completed by December 31, 2025. We anticipate completing the analysis by March 30, 2026.

## Discussion

### Expected Outcomes

We have designed this study based on the foundational concept that this is a behavior change intervention, albeit one where we are measuring medical outcomes. We base the intervention on a specific theory of behavioral change, in this case SCT, to give a framework to the intervention, ensuring clarity about the targets of the intervention and keeping the focus on these targets to avoid scatter-shot ideas about how and where to intervene. We identified a specific and actionable target behavior, walking more, and designed a measurement and feedback system around this focused behavior. This focused approach brings clarity to the researchers and to the patients. The focus lets us determine if this specific intervention increases walking behavior while also assessing any resulting changes in physical parameters such as BMI and ultimately assessing changes in health. It is not sufficient to show that some behavior intervention leads to HbA_1c_ level improvement—we need to understand the steps in between, in particular, the behavior response, to fine-tune the intervention to expand on the aspects that work and eliminate the aspects that do not. We have designed this trial to provide evidence about the intermediate stages.

Our study uses objectively measured data for all primary and many secondary outcomes. The intervention uses wireless technology that allows step count, BW, and BP measurements to be synced automatically to the app. These simple, passive features make for easy-to-use objective measurements, especially for older patients. Daily monitoring of blood glucose level has been shown to have low measurement rates and to not be preferred by patients; therefore, we have not included it in this intervention [57].

We chose HbA_1c_ level as our primary outcome, as it is regarded as both a global standard for measuring glycemic control and an appropriate indicator of the effectiveness of T2D treatment [58]. According to The Japan Diabetes Society’s treatment guideline, patients with T2D should aim to reduce their HbA_1c_ level to <7% to prevent the development of any complications [59]_._ Therefore, we decided to study the proportion of patients who achieved an HbA_1c_ level <7% in addition to studying the absolute change in HbA_1c_ levels.

In this study, we have elected to use a “formidable” control [60] using a fairly high-functioning placebo app. We have initial confirmation of proof of concept from the pilot, and now, we want to show whether our specific behavior change intervention methods are superior to simply giving patients access to their step count, BW, and BP. There is evidence suggesting that simply giving patients a pedometer increases their activity level [61]; thus, there is some risk that the study will induce a response in the control group, which may prevent statistical proof of any superiority of the StepAdd intervention. Most people who want a pedometer already have access to one in their smartphone, and we think this risk is modest. By giving the placebo group a functional app, albeit one lacking StepAdd’s functionality, we have designed the study to give strong proof of the success of StepAdd, if in truth, it is effective.

Many behavior interventions show solid, short-term gains that fade later [62]. Our StepAdd pilot [29] showed results that continued to improve throughout the study, with no sign of fading. This study expands the trial time to 24 weeks with a 12-week observation period to examine whether the pilot results hold true over a long period. Behavior change maintenance is theorized as an outcome of active and ongoing self-regulation, and habit development follows a period of successful self-regulation of a new behavior [63]. Our objective is to change behavior in a sustainable way, such that the new pattern of behavior becomes a habit that can be sustained over the long haul.

A key to our approach is ensuring that the patients receive a strong introduction to the mechanics of the intervention and the StepAdd app. Patients have busy lives, and we work to make it clear and easy for them to meet the demands of the intervention. By providing training regarding the intervention and the app and by ensuring that all messaging in the intervention is simple and focused, we maximize the likelihood that the patients will use the tools of the intervention just as we intend them to.

Another key part of the approach is focusing first on the patients whom the intervention will serve. We designed the intervention for a specific target set, not all patients, and customized the intervention to meet their needs and accommodate their preferences. We have found that it is essential to design with cultural background and patient preference in mind, as we did in the StepAdd pilot [29]. Another key aspect of our approach is applying it only to patients who are already motivated to change their behavior. We filter based on TTM stages, and we have found this approach to be highly effective [29,43]. Our intervention is designed for high-motivation patients who are in need of some help in implementing their desired behavior— low-motivation patients are in need of an entirely different intervention focused on improving their motivation. We have found that large proportions of patients with T2D in Japan have high motivation, with a study showing that 92% are in the contemplation-through-action stages of TTM [64]. There is a very large pool of patients who would benefit from this targeted intervention. Exercise has been shown to improve health in patients with chronic kidney disease [65], and a recent meta-analysis confirms that exercise and diet programs improve the health of patients with T2D who are obese, including improving BMI and HbA_1c_ level [66]. This study is a multicenter randomized controlled trial, and this increases the generalizability of the findings to patients with T2D throughout Japan. Our techniques are general, and there is every reason to believe that these methods could be applied to increase exercise for patients with other diseases and in countries throughout the world.

### Limitations

The study has limitations. The results of our trial may be specific to the population studied and may not be fully generalizable to other populations. Our participants will likely be older people of Japanese ethnicity. There are differences between Japanese and other populations in lifestyles and in the pathophysiology of T2D. The study is limited to patients who are able to use mobile phones; therefore, there may be biases owing to users’ digital literacy. In contrast, results may differ with a young population [67], as it has been reported that young populations are able to complete tasks more successfully on mHealth apps [68] and have high satisfaction compared with their older counterparts [69]. Participants will not be blinded to randomization; therefore, social desirability bias may affect the results. We do not engage on or control for dietary changes, and changes in dietary behavior during the study may affect our results. Baseline step count is measured using pedometers provided to participants, and the resulting measurements may be higher than the true prestudy step count, as studies have found that providing a pedometer increases step count by >2000 steps [63], at least in the short term. This effect is mitigated by our 2-arm trial and the length of our trial, but it may lead us to underestimate the increases in step count and, therefore, overestimate the per-step impact on health outcomes.

This trial will provide important evidence about the efficacy of an SCT-based mHealth intervention in improving physical activities and glycemic control in patients with T2D. If this study proves the intervention to be effective and safe, it could be a key step toward the integration of mHealth as part of the standard treatment received by patients with T2D in Japan. Our findings will inform future studies of using theory-based behavior change techniques and may lead to practical use by exercise professionals in the real world [70].

## Abbreviations

BP: blood pressure
BW: body weight
CONSORT: Consolidated Standards of Reporting Trials
FAS: full analysis set
HbA_1c_: hemoglobin A_1c_
mHealth: mobile health
PPS: per protocol set
SAS: safety analysis set
SCT: social cognitive theory
TTM: transtheoretical model
T2D: type 2 diabetes

## Data Availability

Aggregate data analyzed in this study may be made available upon reasonable request by contacting the corresponding author via the email address provided.
